# Cisplatin Resistant Patterns in Ovarian Cell Line Using FTIR and Principle Component Analysis

**Published:** 2012

**Authors:** Rezvan Zendehdel, Ali Masoudi-Nejad, Javad Mohammadzadeh, Farshad H. Shirazi

**Affiliations:** a *Pharmaceutical Research Center, Shahid Beheshti University of Medical Sciences, Tehran, Iran.*; b *School of Pharmacy, Shahid Beheshti University of Medical Sciences, Tehran, Iran.*

**Keywords:** Drug resistant, Fourier transform infrared, Principle component analysis, Feature extraction, Cell line

## Abstract

Cisplatin is a common chemotherapeutic agent that used for treatment of many solid cancers. Rapid identification of chemotherapy resistance is very important and may lead to effective treatment plan. Spectroscopy techniques, such as infrared spectroscopy, which are sensitive to biochemical composition of samples, have shown potentials to discriminate tissues. Developing in Fourier transform infrared (FTIR) as a diagnostic tool support conventional technique in investigating cell phenotype. By this goal three different cell lines, two cisplatin resistant OV2008-DDP (C13) and A2780-CP ovarian cell lines and one cisplatin sensitive A2780 cell line were investigated by FTIR spectroscopy. Data were subjected to principle component analysis (PCA) to obtain FTIR pattern for cisplatin resistance. Using FTIR spectroscopy on these cells in the range of 400-4000 cm^-1^ was shown dramatic change in cells. Results shows that Cisplatin resistance pattern is characterized in spectrum with the alteration of conformation in secondary structure of proteins and a shift toward the high wave numbers of CH2 stretching vibration. The FTIR data set between 1000 and 3000 cm^-1^ could be consumed as biochemical typicality spectra among resistant and sensitive cell lines while correctly classified by PCA model. Our work supports the promise of PCA analysis of FTIR data as a powerful combined approach for the development of automated methods to recognize resistant to cisplatin in experimental cell lines. One of the advantages of this tool is to investigate the resistant percent of cancer cells .Such technique may bring new tool in cancer diagnosis and stage definition in cancerous tissues.

## Introduction

Cisplatin is a commonly chemotherapeutic agent that used for treatment of many cancer types including testicular, ovarian, cervical, head and neck, non-small cell lung and lymphoma(1). However, resistance to cisplatin therapy is serious problem for successful treatment (2). In ovarian cancers, more than 70% of patients initially respond to therapy with cisplatin; however, this stabilized the five-year survival rate for ovarian carcinoma population less than 25% (3). 

There are many theories to explain platinum resistant activity (4). One of the major mechanisms of resistance to cisplatin is a decreased effective concentration of drug in the cells. Reduction in cisplatin concentration of 20-70% has been observed in cell lines resistant to cisplatin (5). A family of membrane transport proteins has been identified as an implication in this phenomenon. Some reports indicate exposure to cisplatin can cause glycoprotein expression in ovarian cancer cell line (6).

Cisplatin at plasma concentrations down regulates protein expression of copper transporter (Ctr1) in human ovarian carcinoma cell lines (7, 8). In two cell lines, A2780-CP and 2008-C13 over expression of copper transporter ATP7A was shown to be causal for resistance to cisplatin (9). Increased levels of transporter have been observed with decreased lysosomal cellular compartment in the cisplatin-resistant ovarian carcinoma cell line. This cell line also has decreased protein levels of the lysosome associated proteins (10). Although many reporting are about protein content changing in cisplatin resistant cell lines, little is known about structural and conformational protein changing.

There is an increasing interest in the use of FTIR technology in large number of different research. It has been used to investigate biochemical composition of cells (11) and the study of normal and malignant tissue (12-14). These literatures have shown that the FTIR technique can be used to detect a cell phenotype or an illness with a good level of sensitivity.

Molecular difference in sensitive and resistant cell lines by FTIR spectroscopy has not been studied broadly. In this research, we used a FTIR-based assay followed by PCA analysis to look for the resistant specific patterns. This effort demonstrated changes in biochemical index, which can be used for platinum resistant activity. 

## Experimental

*Cell lines*

A2780 (human ovarian carcinoma-sensitive to cisplatin), A2780-CP and C13 (human ovarian carcinoma-resistant to cisplatin) cell lines were obtained from national cell bank of Iran (Tehran, Iran). All cell lines were grown in RPMI-1640 medium and supplemented with 10% heat inactivated fetal bovine serum, antibiotics: penicillin, streptomycin (all chemicals from Sigma, St. Louis, MO, USA). Cells were maintained at 37°C in humidified atmosphere containing 5% CO_2_. The medium was replaced every two day.

*Cell preparation for spectroscopy*

The following procedure was similarly applied for both of sensitive and resistant cell lines. Cells were trypsinized from the original flask and seeded in 25 cm^2^ flasks with fresh medium to reach the logarithmic phase of growth curve. After that cell were washed twice in saline (0.9% NaCl), suspend and centrifuged at 1000 rpm for 5 min, then resuspended in saline to obtain a concentration of 1 × 10^5^ cells. 10 μL of each cell suspension was placed on a zinc selenide sample carrier, which was dehydrated in a vacuum cabin (0.8 bar) for approximately 4 min. These plates were then used for FTIR spectroscopy.

*FTIR spectroscopy*

For FTIR studies, thin dried films of cell suspensions were used on the Zinc selenide window by using a WQF-510 (Rayleigh Optics, China) spectrometer, equipped with a KBr beam splitter and a DLaTGS (deuterated Lantanide triglycine sulphate) detector. The whole system was continuously purged with N2 (99.999%). In each spectrum, 100 scans were collected at a resolution of 4 cm^-1^ for every wave number between 400 and 4000 cm^-1^. These experimental conditions were kept constant for all the measurements. Each single spectrum was baseline corrected and then normalized in order to have the range spanning from 0 to 1.

*Data analysis*

*Data set*

A total of 45 FTIR spectrums between 1000-3000 cm^-1^ have been used in this study as the dataset. Distribution of different FTIR spectra was equal for types of A2780, A2780-CP and C13 cell lines.

*Principle component analysis*

Principle component analysis (PCA) is a well-known method of dimension reduction. The basic idea of PCA is to reduce the dimensionality of a data set, while retaining as much as possible the variation present in the original predictor variables. This is achieved by transforming the *p *original variables *X *= [*x*_1_, *x*_2_,..., *x*_p_] to a new set of *q *predictor variables, *T *= [*t*_1_, *t*_2_,...,*t*_q_], which are linear combinations of the original variables. 

In mathematical terms, PCA sequentially maximizes the variance of a linear combination of the original predictor variables (15). PCA were done using defaults of the program’s parameters. A total of the FTIR output as absorbance percent in different wave number between1000-3000 cm^-1^ have been used for input.

## Result and Discussion

*Spectrum alteration*

**Figure 1:**
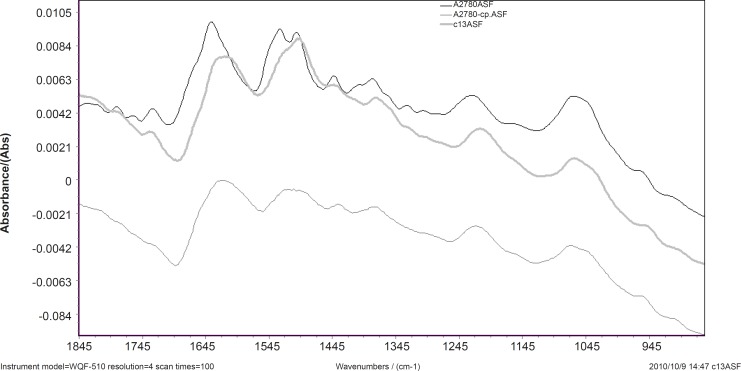
FTIR spectral features of A2780, A2780-CP and C13 cell lines in 1800-900 cm^-1^

Spectral features of A2780, A2780-CP and C13 cell lines are shown in Figure 1 for the region of 1800-900 cm^-1^. The normalized FTIR spectra in this region showed alterations in different spectrum areas. Comparison between spectra showed at least five areas of variation: 

a) there is a peak about 1636 cm^-1^ which can be related to *β*-sheet secondary structure of amid I (16). In the ovarian human resistant cells the peak of about 1636 cm^-1^ shifted toward the lower wave numbers. This may represent the possibility of molecular interaction in *β*-sheet conformation structure of ovarian sensitive cells.

b) The vibration band at 1530 cm^-1^ is assigned to *β*-sheet secondary structure of amid II (17). In A2780-cp cell line; there is a broadband at 1530 cm^-1^ while there are two bands at 1530 and 1540 cm^-1^ in the sensitive cell line. There are positive shoulder peaks at 1540 cm^-1^ and a peak at 1530 cm^-1^ in C13. The bands at 1540 cm^-1^ are assigned to α-secondary structure of amid II (18). This can be showed clear margin of *α*-secondary and *β*-sheet structure of amid II in sensitive cell while in the C13 resistant cell the reduction of *α*-secondary structure of amid II was observed. The result shows *α*-secondary structure conversion to *β*-sheet structure conformation in the C13 resistant cell. 

c) The vibration bands at 1240 cm^-1^ are assigned to amid III (17). In the ovarian human resistant cells the peak of about 1240 cm^-1^ shifted toward the lower wave numbers. This shows the addition of molecular interaction in amid III structure of ovarian sensitive cell. 

d) The vibration band at 1380 cm^-1^ is assigned to glycoprotein (18). There is a peak at this band in the A2780 cell line spectra while in the C13 and A2780-CP cell lines shifted toward the lower wave numbers.

e) There are positive shoulder peaks at 1407 cm^-1^ and 1330 cm^-1^ in the sensitive cell line while there aren’t in the ovarian human resistant cells. 

**Figure 2:**
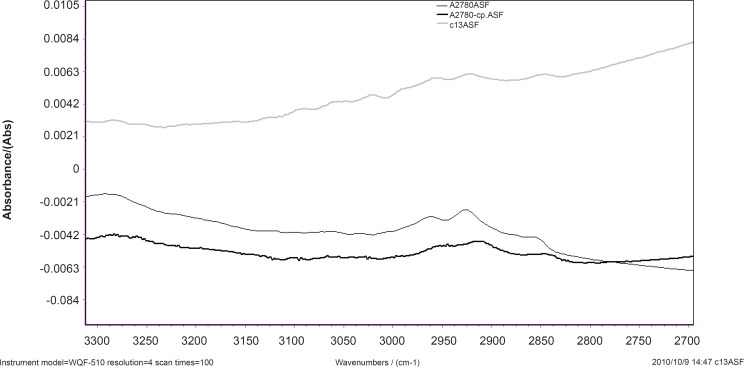
Spectral features of A2780, A27-80-CP and C13 cell lines in 3100-2500 cm^-1^.

The normalized FTIR spectra of A2780, A2780-CP and C13 cell lines are shown in Figure 2 for 3100-2500 cm^-1^. The CH stretching region (3000-2800 cm^-1^) contains the asymmetric and symmetric membrane lipids (20), which are appeared differently in sensitive and resistant cells.

CH_2_ symmetric and asymmetric stretching vibration bands are appeared at 2920 and 2852 cm^-1^ and CH_3_ stretching vibrations at 2950 cm^-1^, respectively (20). Our results show that CH_2 _symmetric and asymmetric stretching vibration bands shift to higher wave numbers in resistant cells. This might be as the result of condensation of the unsaturated lipid cell membrane. In sensitive cell line the intensity of CH_2_ stretching bands and CH_3_ stretching vibrations are higher than resistant cell line. Our research shows that the lipophilicity of cell membrane has been changed in the resistant cells.

*Data processing*

Unsupervised methods such as PCA attempt to explore the data to detect relationships without any presumption on the priority of the various data. These methods identify groups of data elements that are highly correlated with each other. In this study PCA can be used to extract the most significant variations between groups of spectra of cells. Thus, determining an unknown cell type from several possibilities cells can be completed using spectra from known cell types. Score plots provide visualization of the data, whereby the load of data is an indicator of biochemical similarity (21). 

Data of A2780, A2780-cp and C13 cell lines sorted into data sets composed of 45 variables. FTIR wave number from 1000 to 3000 cm^-1^ was used in four segmentation of 1000-1500 cm^-1^ , 1500-2000 cm^-1^, 2000-2500 cm^-1^ and 2500-3000 cm^-1^. There are no suitable clustering with PCA for four segmentation of data set (Data was not shown).These result show, the lipid stretching region (2500-3000 cm^-1^) and protein band (20) region (1500-2000) could not discriminate the cell type.

**Figure 3:**
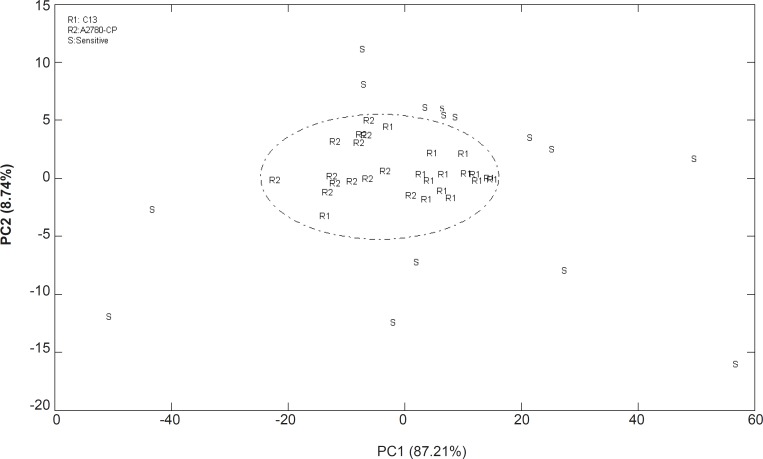
Score plot of PCA analysis for the FTIR spectral data of A2780, A2780CP and C13 cells in the region of 900 to 1500 cm^-1^(R: resistant and S: sensitive

The data of the cell lines between 1000 and 3000 cm^-1^ were analyzed with PCA resulting in the clustered shown in Figure 3.

 The cluster of points derived from the first two PC scores which summarized spectral features of three cell lines are shown in a 2-dimensional projection. PCA correctly classified 96% of all spectra using two PCs and thus provide a good separation for representing the variety of sensitive and resistant cell line spectra. The data of the resistant cells are in the central area of PCA projection while it could not be distinguished between resistant cells. There are similarities between the resistance cell lines of A2780-CP and C13 spectrum, which could not discriminate using this technique. Thus the molecular compositions of two different resistant cell lines which cause resistance to cisplatin might almost be similar.

**Figure 4:**
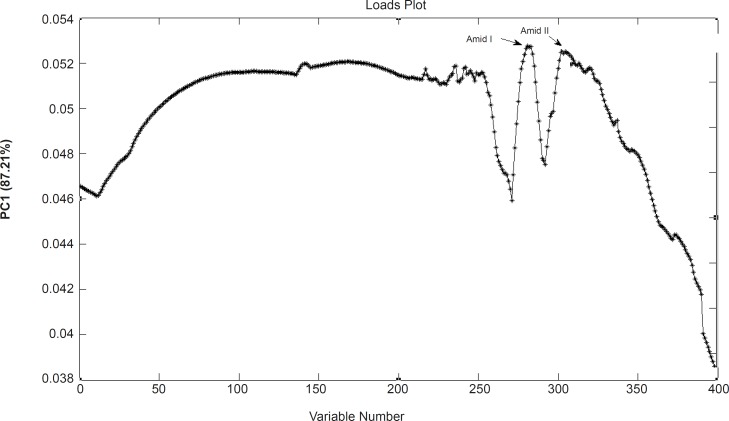
Loading plot of PCA analysis as is resulted from the FTIR data of A2780, A2780CP and C13 cells

Loadings plots allow for identification of wave numbers and corresponding biochemical responsibility. Figure 4 shows the loading plot of PC1 resulted from the FTIR spectra of these cell lines. This figure clearly shows that the variables from of Amid I and Amid II bands (18) strong loadings contributing to the separation of sensitive and resistant cell lines. 

## Conclusion

Resistance to Chemotherapy is a serious hurdle in the treatment plan of cancer. That is why identification of resistant cells at the procedure of a patient treatment saves time and is very much critical for the therapy outcome. The FTIR spectra provide a unique molecular fingerprint that can be interpreted based on the macromolecular biochemistry of the cells. This study suggests that infrared spectroscopy and clustering of data with PCA may represent a simple and precise method for discrimination biochemical pattern of resistant in cell lines. One of the advantages of this tool is to investigate the resistant percent of cancer cells using FTIR. Such technique may bring new tool in cancer diagnosis and stage definition in cancerous tissues. The FTIR data set between 1000 and 3000 cm^-1^ in resistant and sensitive cells correctly classified by PCA model This region of FTIR data could be consumed as “Biochemical typicality spectra” among resistant and sensitive cell lines. In this region amid I and amid II molecules band have higher difference in sensitive and resistant cells.
